# Squamous cell carcinoma of the sigmoid colon: a rare case report

**DOI:** 10.1093/jscr/rjaf607

**Published:** 2025-12-04

**Authors:** Sadiq Husain, Mohammad Aslam

**Affiliations:** Department of Surgery, Aligarh Muslim University, Aligarh 202002, UP, India; Department of Surgery, Aligarh Muslim University, Aligarh 202002, UP, India

**Keywords:** squamous cell carcinoma, sigmoid colon, colorectal cancer, rare malignancy, intestinal obstruction, FOLFOX, case report

## Abstract

Squamous cell carcinoma (SCC) of the colorectum is rare malignancy. It constitutes only 0.1–0.25 per 1000 cases of colorectal carcinoma. Here, we report a rare case of primary SCC of the sigmoid colon in a 40-year-old female from Western Uttar Pradesh, India. She presented with acute intestinal obstruction and a history of altered bowel habits and decreased appetite. Surgical resection revealed a constricted tumor in the sigmoid colon. Histopathological analysis confirmed the diagnosis of SCC with no lymph node involvement (0/32). The patient received four cycles of adjuvant chemotherapy (FOLFOX regimen) and has remained disease-free during a 3-year follow-up. Given its rarity, there is no established treatment protocol for colorectal SCC. Surgical resection remains the mainstay of treatment, with uncertain benefits from chemotherapy or radiotherapy. Early diagnosis and long-term follow-up are crucial for favorable outcomes in such uncommon presentations.

## Introduction

Carcinoma of the colorectum is a common malignancy. Adenocarcinomas constitute the majority of cases. The incidence of squamous cell carcinoma (SCC) of the colon and rectum has been reported to be 0.25 to 0.1 per 1000 cases of colorectal carcinoma [1, 2]. Only a few hundred cases have been reported in the literature to date. Here, we report a case of SCC of the sigmoid colon in a middle-aged female from Western Uttar Pradesh.

## Case presentation

**Figure 1:**
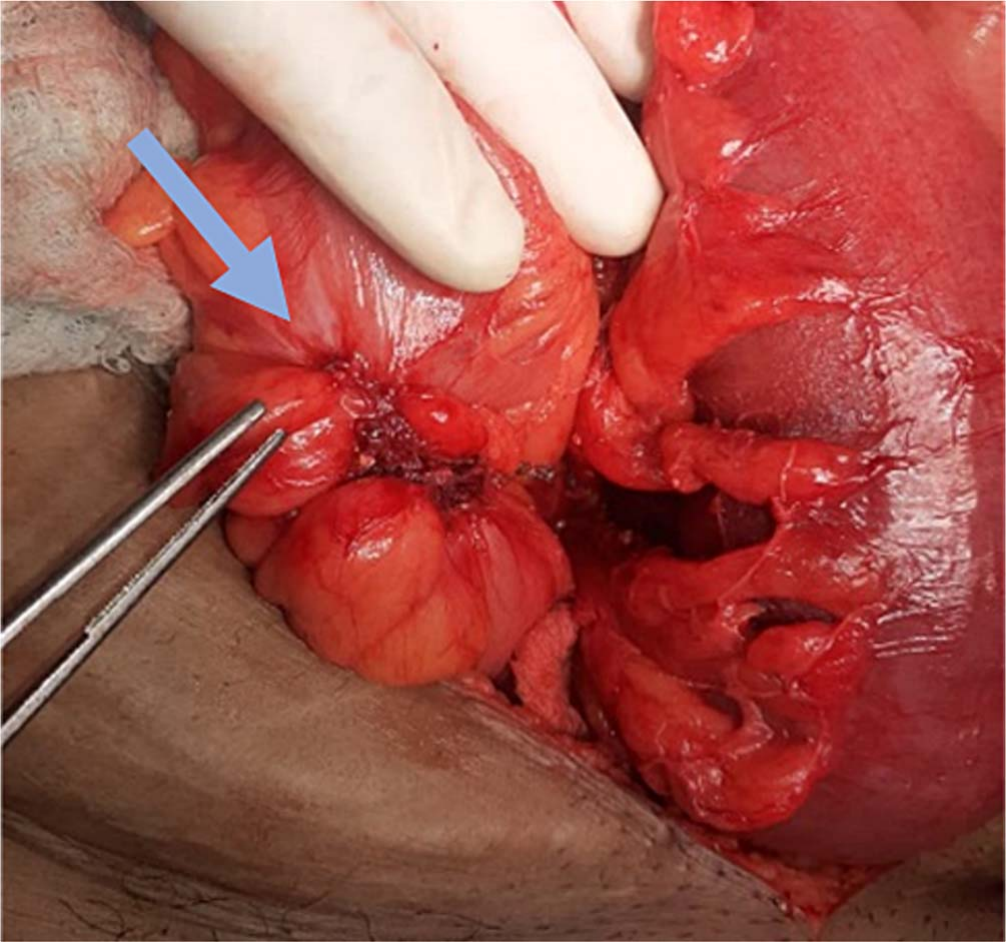
Sigmoid colon tumor (arrow).

A 40-year-old female, the mother of three children, presented to the emergency department with non-passage of stool or flatus for 5 days, and vomiting and abdominal distension for 4 days. The patient had altered bowel habits and a decreased appetite for the last 2 months at which point she was taking remedies at home. There was no history of bleeding per rectum. She was thin and anemic. Her abdomen was distended, and her bowel sounds increased in intensity and frequency. No growth/lesions were appreciable on rectal examination. The patient underwent surgery for intestinal obstruction, and constricted growth was found in the sigmoid colon, which was ~3 cm in length and did not adhere to the parietal wall or other bowel loops (Fig. 1). No other lesions were appreciable in the bowel, liver, peritoneum, or mesentery.

The involved segment of the sigmoid colon was resected at 5 cm margins both proximally and distally to the growth, and a sigmoid colostomy with a distal mucus fistula was created.

**Figure 2:**
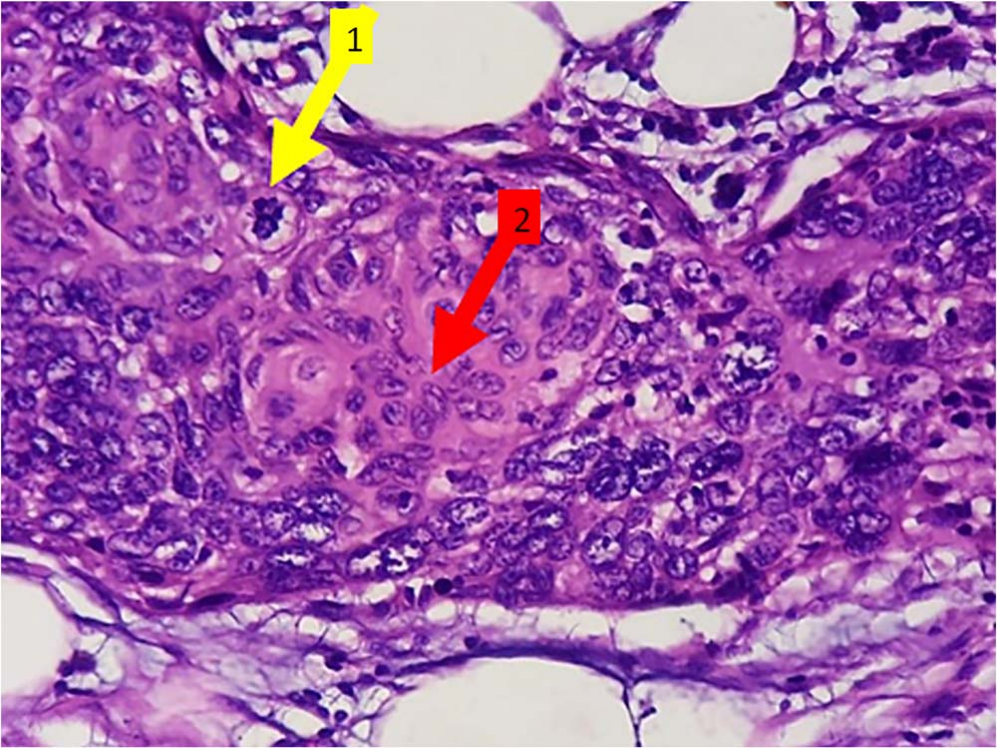
(H&E 400×) Showing malignant squamous cells with increased N:C ratio, nuclear pleomorphism, vesicular to hyperchromatic nuclei, and occasional nucleoli. Evidence of mitotic figures (arrow marked 1) and keratinization (arrow marked 2).

Histopathology revealed SCC of colon (Fig. 2). The lymph nodes were not involved (0/32).

The patient was given four cycles of adjuvant chemotherapy (FOLFOX regimen) and was under regular follow-up. The patient has no evidence of disease recurrence even after 3 years.

## Discussion

Colorectal cancer (CRC) is the third most common cause of cancer-related death worldwide [3]. Among all cases of CRC ~90% are adenocarcinomas, whereas carcinomas, sarcomas, and lymphoid tumors constitute the remaining 10% of cases [4].

SCC in the gastrointestinal tract is a rare condition, and its occurrence in the colorectum is extremely rare, reported to be almost 0.1–0.25 per 1000 cases of CRC [1, 2].

Adenosquamous carcinoma was reported for the first time by Herxheimer in the cecum, but the first case of pure SCC of the colon was reported by Schmidtmann in 1919 in a 65-year-old man [5]. The first case of pure SCC of the colon in India was reported by Bhat *et al*. in a 55-year-old female from the southern part of the country [6]. As of 2010, almost 120 cases of SCC had been reported worldwide [7].

In 1979, Williams *et al*. formulated certain criteria that must be fulfilled to diagnose primary SCC of the colorectum [8]. It includes (A) the absence of evidence of SCC of any other part of the body, ruling out any chance of possible metastasis from any organ to the colorectal site; (B) the exclusion of any proximal extension of anal SCC; (C) the absence of a fistulous tract lined by squamous cells; and (D) the confirmation of SCC by histological analysis.

SCC of the colorectum affects individuals with a mean age of 55–60 years, and women are more frequently affected with SCC colorectum (66% of cases are reported in women) [7]. Juturi *et al*. reported a slight male predominance (59% of 34 cases of colorectal SCC) [9]. Data regarding the prognosis of this disease are limited because of the rare nature of this malignancy.

The clinical symptoms of SCC of the rectum are the same as those of a typical adenocarcinoma: pain, tenesmus, rectal bleeding, and weight loss [10–12]. As in adenocarcinoma, the duration of symptoms is highly variable and ranges from several weeks to many months. Our patient presented to the hospital for the first time with acute intestinal obstruction, although she had altered bowel habits and a decreased appetite for the last 3 months.

Establishing a management protocol for the SCC colon is difficult because only a few cases have been reported that were followed up for a long period. However, management approaches reported in the literature provide few insights. The most definitive curative potential remains in the surgical resection for most patients. The role of neoadjuvant or adjuvant radiotherapy or chemotherapy has yet to be established. Chemotherapy alone or in combination with radiation therapy and/or surgery has been described [10, 13, 14].

Wu *et al*. administered four cycles of neoadjuvant oxaliplatin and capecitabine (XELOX) before right hemicolectomy, and postoperative paclitaxel/cisplatin (TC) adjuvant chemotherapy. However, after 23 months, patient presented with abdominal metastasis [15].

In a systematic review of 99 patients with SCC colon cancer by Schizas *et al*., the 1- and 3-year survival rates were reported to be 81.2% and 54.2%, respectively, whereas the 5-year survival rate was 49.5% [16].

## Conclusion

Carcinoma in the colorectal region is common, and almost all cases are adenocarcinomas. The occurrence of SCC in this region is unusual, and only a few cases are available in the literature. The presenting symptoms are similar to those of adenocarcinoma colon, but females are found to have this variant of CRC more frequently than males. The prognosis of SCC is poorer than that of adenocarcinoma of the colorectal region. As this disease is uncommon, specific management protocol has yet to be established.
